# Renewable Energy Consumption and Economic Growth in Nine OECD Countries: Bounds Test Approach and Causality Analysis

**DOI:** 10.1155/2014/919167

**Published:** 2014-01-14

**Authors:** Lin Hung-Pin

**Affiliations:** Department of International Business & Trade, Shu-Te University, No. 59, Hun Shan Road, Yen Chau, Kaohsiung 82445, Taiwan

## Abstract

The purpose of this paper is to investigate the short-run and long-run causality between renewable energy (RE) consumption and economic growth (EG) in nine OECD countries from the period between 1982 and 2011. To examine the linkage, this paper uses the autoregressive distributed lag (ARDL) bounds testing approach of cointegration test and vector error-correction models to test the causal relationship between variables. The co-integration and causal relationships are found in five countries—United States of America (USA), Japan, Germany, Italy, and United Kingdom (UK). The overall results indicate that (1) a short-run unidirectional causality runs from EG to RE in Italy and UK; (2) long-run unidirectional causalities run from RE to EG for Germany, Italy, and UK; (3) a long-run unidirectional causality runs from EG to RE in USA, and Japan; (4) both long-run and strong unidirectional causalities run from RE to EG for Germany and UK; and (5) Finally, both long-run and strong unidirectional causalities run from EG to RE in only USA. Further evidence reveals that policies for renewable energy conservation may have no impact on economic growth in France, Denmark, Portugal, and Spain.

## 1. Introduction

Ongoing debate on whether and how energy consumption affects economic growth has been an extensive public topic of discussion in the industrialized world. To address this issue, delegates from more than 160 countries met in Kyoto, Japan, in 1997, to draft an agreement called the Kyoto Protocol. This agreement demands a decrease in carbon dioxide emissions. The US tends to not sign any protocol that fails to include binding targets and timetables for both developing and industrialized nations or a protocol that would seriously harm its economy. This leads to a dilemma regarding whether the policy focus should be on energy saving and carbon reduction or economic growth. Many studies have discussed the relation between energy consumption and economic growth by using various methodologies for different time periods and countries since the study of J. Kraft and A. Kraft [1], who found the evidence of causal relation between energy consumption and GNP in the US. Existing studies on this topic can be classified into four types. (1) Growth hypothesis: this first type includes Akinlo [2], Ho and Siu [3], Narayan and Singh [4], Shiu and Lam [5], and Yuan et al. Reference [6] indicates that unidirectional causality runs from energy consumption to economic growth. This growth hypothesis also implies that the decrease in energy consumption due to energy conservation-oriented policies may deteriorate economic growth. (2) Conservation hypothesis: this second type comprising Ghosh [7], Jumbe [8], Halicioglu [9], Narayan and Smyth [10], and Yoo and Kim [11] suggests that unidirectional causality runs from economic growth to energy consumption. This conservation hypothesis also implies that energy conservation policies designed to decrease energy consumption may have no impact on economic growth. (3) Feeback hypothesis: the third type which includes Narayan and Smyth [12], Oh and Lee [13], Yang [14], and Yoo [15] suggests that there is a bidirectional causality between economic growth and energy consumption. (4) Neutrality hypothesis: Wolde-Rufael [16] and Yoo [17] which belong to the fourth type indicate the absence of a causal relationship between economic growth and energy consumption.

According to the above debates, admittedly, the available evidence between energy consumption and economic growth is inconclusive and they do not indicate the long-run effects of energy consumption on economic growth as a whole. The purpose of this paper is to investigate the causality between renewable energy consumption and economic growth and to derive policy implications from the empirical results. We conduct both short-run and long-run renewable energy consumption impact on economic growth and, to the best of our knowledge, it is the first to examine this issue in OECD countries. To this end, we use a different approach to investigate the effect of renewable energy consumption on economic growth—namely, an autoregressive distributed lag (ARDL) cointegration test developed by Pesaran et al. [18] and Granger causality analysis for these nine OECD countries, Denmark, France, Germany, Italy, Japan, Portugal, Spain, UK, and USA based on the data from the period between 1982 and 2011. These countries were chosen because they are among the largest renewable energy using in OECD countries. The region's rapidly rising renewable energy demand (7.6% per year) reflects both the environmental awareness of the public and the higher per capital renewable energy consumption rates, particularly in USA and Germany. Overall, these countries' per capital renewable energy consumption almost doubled from 4,660 GWh to 8,942 GWh during the period. This reveals an average annual growth rate of 11.3%, the first most rapid in the world comparing to the other regions.

Due to the mentioned developments in renewable energy consumption and that limited studies had been stressed in this subject for these selected OECD countries, the message of this paper is all the most useful, because it is the first empirical study that explores whether or not the causalities between renewable energy consumption and economic growth exist in OECD countries. The results obtained in this paper are related by the sample period, the variables used, and the methodology conducted. The rest of this paper is organized as follows.  Section 2 constructs the estimation procedure including ADF test, ARDL approach, and Granger causality analysis.  Section 3 explains the empirical results. Finally, concluding remarks and policy implications are presented in Section 4.

## 2. Methodology and Data

### 2.1. Unit Root Test: Augmented Dickey Fuller (ADF)

The annual time series data of renewable energy consumption for Denmark, France, Germany, Italy, Japan, Portugal, Spain, UK, and USA are measured in quadrillion Btu and obtained from Energy Information Agency and International Energy Agency (Energy Statistics of OECD Countries) (renewable energy source represents energy consumption related to biomass, geothermal, hydroelectric power, solar, and wind power). The economic growth (Real GDP) data is sourced from the Databanks of World Development Indicates (WDI). The assumptions of the classical time series model require that series {*x*_*t*_} is stationary and errors have a zero mean and finite variance. Nonstationary variables may result in a spurious regression if the nonstationary properties of the variables are not reflected (Granger and Newbold [19]). Therefore, unit root test is applied to determine whether the variables are stationary individually before conducting causality tests. Numerous macroeconomic time series contain unit roots dominated by stochastic trends as developed by Nelson and Plosser [20]. Unit roots are crucial in examining the stationarity of a time series because a nonstationary regressor can invalidate standard empirical results. The presence of a stochastic trend is determined by testing for the presence of unit roots in time series data. In this paper, a unit root test is tested by using Augmented Dickey-Fuller (ADF).

The Augmented Dickey-Fuller test [21] is referred to by the *t* statistic of *λ*_2_ coefficient of the following regression:

(1) $$\begin{array}{c} \Delta x_{t}=\lambda_{0}+\lambda_{1}t+\lambda_{2}x_{t-1}+\sum_{i=1}^{n}\eta_{i}\Delta x_{t-1}+\mu_{t}, \end{array}$$

where Δ  is the first difference operator with *n* lags, and *μ*_*t*_ is a stationary random error which adjusts the error of autocorrelation. The null hypothesis is that *x*_*t*_ is a nonstationary series and rejected when *λ*_2_ is significantly negative (*H*_0_ : *λ*_2_ = 0; *H*_1_ : *λ*_2_ < 0). This paper uses the Schwarz-Bayesian criteria (SBC) to determine the optimal lag orders for (1) by selecting the grid of values for the number of lags (*n*) and obtaining the value of *n* at which the SBC attains its minimum.

### 2.2. Autoregressive Distributed Lag (ARDL) Cointegration Tests

The standard log-linear function of long-run relationship between renewable energy consumption and real GDP per capita in selected nine OECD countries can be expressed as

(2) $$\begin{array}{c} Y_{t}=\beta +\delta \text{R}{\text{E}}_{t}+\mu_{t}, \end{array}$$

where RE_*t*_ is renewable energy consumption (GWh per capita), *Y*_*t*_ stands for real GDP per capita which can be denoted by economic growth, and *μ*_*t*_ is the usual error term. All variables are taken in their natural logarithms prior to conducting the empirical analysis. We test for the existence of a long-run cointegrating relationship between renewable energy consumption (RE_*t*_) and real GDP per capita (*Y*_*t*_) by using the bounds test of autoregressive distributed lag (ARDL) approach developed by Pesaran et al. [18]. There are two reasons for applying ARDL approach in this paper. First, the ARDL is applicable irrespective of whether the considered variables are *I*(0) or *I*(1) or a mixture of both, stationary or nonstationary, and thus avoids the spurious regression or problems inherent in the unit root test prior to testing for cointegration. Second, using the ARDL approach avoids a low power in detecting the cointegrating relationship, while the sample or data span is inevitably small (Narayan [22]).

The error correction model (ECM) of the ARDL model is expressed as

(3) ΔYt=β1+∑i=1m1θ1iΔYt−i+∑j=0n1δ1jΔREt−j+γ1Yt−1+γ2REt−1+μt,

where Δ is the first difference operator. In (3), the null hypothesis of the cointegrating relationship between *Y*_*t*_ and RE_*t*_ is detected by testing the *F*-statistic for *H*_0_ : *γ*_1_ = *γ*_2_ = 0 against the alternative *H*_1_ : *γ*_1_ ≠ *γ*_2_ ≠ 0. Instead of the conventional critical values, Pesaran et al. [18] proposed a bounds test for two sets of critical variables. The first set assumes that all variables are *I*(0), and the other set assumes that all variables are *I*(1). If the tested *F*-statistic value lies below the lower bound critical value, then the null hypothesis of no cointegrating relationship cannot be rejected, and if it exceeds the respective upper bound critical value, the null hypothesis is rejected. If the tested *F*-statistic value falls within the lower and upper critical value bounds, inference is inconclusive. The set of the bound critical values for the limited data was recently developed by Narayan [22].

If there is a cointegrating relationship between variables, the next step is to estimate the following long-run model and short-run dynamics in (4) and (5), respectively,

(4) $$\begin{array}{c} \Delta Y_{t}=\beta_{2}+\sum_{i=1}^{m2}\theta_{2i}\Delta Y_{t-i}+\sum_{j=0}^{n2}\delta_{2j}\Delta \text{R}{\text{E}}_{t-j}+\mu_{2t}, \end{array}$$

(5) $$\begin{array}{c} \Delta Y_{t}=\beta_{3}+\sum_{i=1}^{m3}\theta_{3i}\Delta Y_{t-i}+\sum_{j=0}^{n3}\delta_{3j}\Delta \text{R}{\text{E}}_{t-j}+\phi \varepsilon_{t-1}+\mu_{3t}, \end{array}$$

where *ϕ* is a statistically significant coefficient of error correction term (*ε*) with a negative sign and shows how fast variables converge to the equilibrium.

### 2.3. Causality Tests

The ARDL approach reveals the long-run cointegration information, but it does not indicate causal relationship between variables. Thus, the two-step procedure of Engle and Granger [23] causality test is conducted by examining the causal relationship between the renewable energy consumption and economic growth. To investigate the short-run and long-run Granger causality relationship, we estimate (4) to obtain the estimated residuals and then employ the following error correction model:

(6) $$\begin{array}{c} \Delta Y_{t}=\beta_{4}+\sum_{i=1}^{m4}\theta_{4j}\Delta Y_{t-i}+\sum_{j=0}^{n4}\delta_{4i}\Delta \text{R}{\text{E}}_{t-j}+\phi_{1}\varepsilon_{t-1}+\mu_{4t}, \end{array}$$

(7) $$\begin{array}{c} \Delta Y_{t}=\beta_{5}+\sum_{i=0}^{m5}\theta_{5j}\Delta Y_{t-i}+\sum_{j=1}^{n5}\delta_{5i}\Delta \text{R}{\text{E}}_{t-j}+\phi_{2}\varepsilon_{t-1}+\mu_{5t}, \end{array}$$

where both residual terms *μ*_4*t*_ and *μ*_5*t*_ are independently and normally distributed with zero mean and constant variance, and the SBC is used to select the optimal lag structure for ARDL specification. If it rejects the null hypothesis, it implies that RE does Granger cause *Y*, and *Y* does Granger cause RE, respectively. Granger causal relationship can be conducted in three ways by using (6) and (7).

1. Short-run Granger causality is conducted by testing *H*_0_ : *δ*_4*i*_ = 0 and *H*_0_ : *θ*_5*j*_ = 0 for all *i* and *j*, respectively.
2. Long-run Granger causality is conducted by testing *H*_0_ : *ϕ*_1_ = 0 and *H*_0_ : *ϕ*_2_ = 0 and notes that the coefficient *ϕ* of *ε* measures how fast the deviations from the long-run equilibrium are shrunk following changes of each variable.
3. Strong Granger causality is detected by testing *H*_0_ : *δ*_4*i*_ = *ϕ*_1_ = 0 and *H*_0_ : *θ*_5*j*_ = *ϕ*_1_ = 0 for all *i* and *j*, respectively.

## 3. Empirical Results and Discussion

### 3.1. Results of Unit Root Tests

ADF unit root test is our first step to confirm the stationary and the degree of integration of each variable. If the order of integration of any of the variables is larger than one, then the critical bounds suggested by Pesaran et al. [18] are not valid. They are only computed on the basis that variables are *I*(0) or *I*(1). The ADF test results are presented in Table 1 for the level term and the first difference of each of the variables. Table 1 shows that the renewable energy consumption variable for Portugal and the economic growth variable for Denmark and Portugal are nonstationary both in level terms and the first differences. This result suggests that we should drop Denmark and Portugal for renewable energy consumption and economic growth nexus from the ARDL bounds testing approach of cointegration and causality analysis because of the different integration orders. Beside that, we can confidently employ the ARDL bounds approach to our model for other countries.

### 3.2. Results of ARDL Cointegration Tests

According to an optimal lag for ARDL model selected by SBC (Pesaran and Shin, [24]), Table 2 shows the estimated ARDL model that has passed several diagnostic tests which indicate no evidence of serial correlation and heteroskedasticity. The bounds *F*-statistic for cointegration test reports a long-run relationship between renewable energy consumption and economic growth at 1% significance level for Japan and UK, 5% significance level for Italy, and 10% significance level for USA and Germany. On the other hand, the ARDL bounds test results show that there is no long-run or equilibrium relationship between renewable energy consumption and economic growth in France and Spain. Thus, cointegration and the causal relationships within dynamic VEC model for France and Spain cannot be estimated.

### 3.3. Results of Causality Tests

The existence of an ARDL cointegration relationship between renewable energy consumption and economic growth for Germany, Italy, Japan, UK, and USA provides that there should be Granger causality in at least one direction. The causality test results in Table 3 for Germany, Italy, Japan, UK, and USA are as follows.

1. There is a short-run unidirectional Granger causality running from real GDP to renewable energy consumption in Italy and UK.
2. There is a long-run unidirectional Granger causality running from renewable energy consumption to real GDP in Germany, Italy, and UK.
3. There is a long-run unidirectional Granger causality running from real GDP to renewable energy consumption in USA and Japan.
4. There exist both long-run and strong unidirectional Granger causalities running from renewable energy consumption to real GDP for Germany and UK.
5. Finally, there exists both long-run and strong unidirectional Granger causalities running from real GDP to renewable energy consumption in only USA.

## 4. Conclusion

This paper attempts to investigate the causal relationship between renewable energy consumption and economic growth for renewable energy-using countries of OECD—Denmark, France, Germany, Italy, Japan, Portugal, Spain, UK, and USA. We use the ADF test to confirm the stationary and integration orders of variables and then the two-step procedure of Engle and Granger model is applied in this paper. In first step, the ARDL bounds testing approach of cointegration is conducted to explore the long-run relationship between the renewable energy consumption and economic growth for each country. Secondly, a dynamic error-correction model is employed to test the causal relationship between these variables.

The main findings of this paper are as follows. (1) The unit root tests results show that variables for Denmark and Portugal do not meet the underlying assumptions of the ARDL bounds testing of cointegration before proceeding to further estimation. Thus, we drop these two countries from the ARDL bounds testing of cointegration and causality analysis. (2) No unique long-run or equilibrium relationship exists between renewable energy consumption and economic growth in France and Spain; hence, any causal relationships within dynamic error-correction model in these two countries cannot be estimated. (3) there is a short-run unidirectional Granger causality running from real GDP to renewable energy consumption in Italy and UK. (4) There is a long-run unidirectional Granger causality running from renewable energy consumption to real GDP in Germany Italy, and UK. (5) There is a long-run unidirectional Granger causality running from real GDP to renewable energy consumption in USA and Japan. (6) There exist both long-run and strong unidirectional Granger causalities running from renewable energy consumption to real GDP for Germany and UK, and, finally, there exist both long-run and strong unidirectional Granger causalities running from economic growth to renewable energy consumption in only USA.

The empirical results of this paper provide policy makers a better understanding of renewable energy consumption—economic growth nexus to formulate investment policies in OECD countries. To practically concern about the environmental awareness and the increased demand for renewable energy that accompanies rapid economic growth, policy makers should endeavor to uncover the causal relationship between renewable energy consumption and economic growth and to formulate appropriate renewable energy policies. This task has become their top priority in the present and near future. Therefore, as a policy implication, USA, Japan, Germany, Italy, and UK could promote their investment on renewable energy infrastructure or regulate the renewable energy conservation policies to avoid a possibility of reduction in renewable energy consumption adversely affecting economic growth. In addition, in USA, the long-run and strong causality evidence may reveal that supporting various energy tax preferences or financial incentives of energy-saving utilization to promote both renewable energy consumption and economic growth is a feasible policy. Further evidence suggests that policies for renewable energy conservation may have little or no impact on economic growth in the remaining four countries, France, Denmark, Portugal, and Spain.

## Figures and Tables

**Table 1:** ADF unit root test results.

Country	Renewable energy consumption		Real GDP per capita	
	Level term	First differences	Level term	First differences
Denmark	−0.108 (1)	−5.331 (0)	−1.098 (0)	−1.331 (0)*
France	−1.432 (0)	−6.509 (0)	−0.132 (1)	−3.887 (0)
Germany	−1.880 (0)	−8.009 (0)	−2.145 (1)	−7.542 (0)
Italy	−1.431 (0)	−3.114 (0)	−1.221 (0)	−7.009 (0)
Japan	−1.527 (0)	−4.772 (0)	−1.551 (0)	−5.760 (0)
Portugal	−0.764 (0)	−0.321 (0)*	−1.423 (2)	−1.654 (0)*
Spain	−0.662 (0)	−4.092 (0)	−2.887 (1)	−3.061 (0)
UK	−3.632 (0)	−5.521 (0)	−2.901 (0)	−4.321 (0)
USA	−1.446 (0)	−4.144 (0)	−2.112 (0)	−4.433 (0)

Notes. First, all the regressions include an intercept and a linear trend in the levels and include an intercept in the first differences; second, numbers in parentheses are the optimal lag orders and selected based on Schwarz Bayesian; and finally *represents the 10% significance level.

**Table:** (a)

Countries	*F* ^a^	LM^c^	HET^d^
France	0.03	0.66 (0.45)	0.23 (0.91)
Germany	3.49*	0.68 (0.34)	0.81 (0.92)
Italy	5.66**	3.95 (0.22)	0.34 (0.56)
Japan	8.12***	0.78 (0.43)	0.28 (0.76)
Spain	1.62	2.04 (0.11)	0.56 (0.72)
UK	9.81***	0.33 (0.52)	2.45 (0.46)
USA	3.18*	0.46 (0.51)	3.18 (0.05)

**(b):** 

Asymptotic critical values^b^					
1%		5%		10%	
*I*(0)	*I*(1)	*I*(0)	*I*(1)	*I*(0)	*I*(1)
6.027	6.76	4.09	4.663	3.303	3.797

Notes. First, *F* is the ARDL co-integration test; second, the asymptotic critical value bounds are obtained from Narayan (2005, Appendix: Case II); third, LM is the Lagrange multiplier test for serial correlation with a *x* ^2^ distribution with only one degree of freedom; forth, HET is the test for heteroskedasticity with a *x* ^2^ distribution with only one degree of freedom; and finally, ***, **, and * represent the 1%, 5%, and 10% significance level, respectively.

**Table 3:** Granger causality test results.

Null hypotheses	USA	Japan	Germany	Italy	UK
*F*-statistics for short-run Granger causality					
Δ(RE)⇒Δ(*Y*)	1.472	0.712	3.604	0.762	0.143
(*H*_0_ : *θ*_4*j*_ = 0)	(0.244)	(0.532)	(0.541)	(0.509)	(0.904)
Δ(Y)⇒Δ(RE)	2.641	0.126	1.877	3.188	3.578
(*H*_0_ : *δ*_5*j*_ = 0)	(0.756)	(0.683)	(0.384)	(0.041)**	(0.064)**

*F*-statistics for long-run Granger causality					
Δε⇒Δ(*Y*)	0.274	3.574	17.571	13.849	7.102
(*H*_0_ : *ϕ*_1_ = 0)	(0.856)	(0.493)	(0.002)***	(0.060)**	(0.013)**
Δε⇒Δ(RE)	11.643	14.744	0.539	3.567	0.612
(*H*_0_ : *ϕ*_2_ = 0)	(0.001)***	(0.001)***	(0.676)	(0.211)	(0.331)

*F*-statistics for strong Granger causality					
Δ(RE), Δε⇒Δ(*Y*)	2.116	0.512	10.667	2.303	2.680
(*H*_0_ : *θ*_4*j*_ = *ϕ*_1_ = 0)	(0.563)	(0.361)	(0.004)***	(0.379)	(0.031)**
Δ(Y), Δε⇒Δ(RE)	9.224	4.153	1.990	2.754	1.564
(*H*_0_ : *δ*_5*j*_ = *ϕ*_2_ = 0)	(0.001)***	(0.142)	(0.348)	(0.218)	(0.452)

Notes. First, (RE) is the renewable energy consumption, (*Y*) is the real GDP per capita, and Δ is the first difference operator; second, *** and ** represent the 1% and 5% significance level, respectively; third, the number inside the parenthesis is the *P*-value for *F*-statistics; and finally, the numbers of optimal lags are selected based on SBC and calculated as one for Japan and UK, and two for USA, Germany, and Italy.
